# Comparing physical education course satisfaction between students with hearing and visual impairments in Mainland China: a revised importance—Performance analysis

**DOI:** 10.3389/fpsyg.2026.1895953

**Published:** 2026-09-29

**Authors:** Lihua Cui, Wenpeng Zhan

**Affiliations:** 1 Department of Physical Education, Yong In University, Yongin-si, Republic of Korea; 2 Department of Social Sports Guidance and Management, Lingnan Normal University, Zhanjiang, China

**Keywords:** adaptive physical education, revise importance-performance analysis, satisfaction, sensory disabilities, visual impairments and blindness

## Abstract

**Introduction:**

This study examined physical education satisfaction among Chinese university students with hearing and visual impairments using Revised Importance-Performance Analysis (RIPA).

**Methods:**

A cross-sectional survey was conducted with 449 students from 10 universities in Guangdong Province, China, including 251 students with hearing impairments and 198 students with visual impairments. The study assessed perceptions of instructional content, teaching practices, learning outcomes, and overall physical education experiences.

**Results:**

The findings revealed distinct patterns of needs and satisfaction between the two groups. Students with hearing impairments placed greater importance on instructional clarity, communication accessibility, and individualized support, whereas students with visual impairments prioritized course accessibility, activity safety, and support for sustained physical activity engagement. Across both groups, immediate participation experiences and inclusive learning environments were valued more highly than long-term physical fitness outcomes.

**Discussion:**

These findings indicate that physical education satisfaction among students with sensory impairments is shaped by disability-specific needs and educational experiences. Accordingly, universities should adopt disability-specific physical education strategies by strengthening communication accessibility and individualized instructional support for students with hearing impairments, while emphasizing activity accessibility, safety, and sustained physical activity engagement for students with visual impairments.

## 1 Introduction

Within the policy framework of *Health First* and educational equity in mainland China, physical education for university students with hearing and visual impairments is an integral component of school physical education and higher special education. National policies emphasize incorporating students' physical health into educational evaluation, promoting rehabilitation and fitness for individuals with disabilities, and expanding inclusive and accessible higher education with strengthened support for students with sensory impairments (General Office of the State Council of the People's Republic of China, 2019, 2021). These initiatives position equitable access to physical education and health-promotion resources as an important responsibility of higher education.

Learning satisfaction reflects students' evaluations of how well course design, instructional interaction, assessment, and support resources meet their learning needs. For students with hearing and visual impairments, it also reflects whether physical education provides an accessible and supportive learning environment. According to Self-Determination Theory, environments that support autonomy, competence, and relatedness promote intrinsic motivation and positive learning experiences (Ryan and Deci, 2000). However, the conditions supporting these needs may differ across sensory impairment groups. For students with hearing impairments, communication accessibility and instructional support are particularly relevant; visualized communication, responsive interactions, clear demonstrations, and adaptive communication strategies can facilitate participation and engagement (Kurková and Scheetz, 2016; Rich et al., 2024). In contrast, students with visual impairments may encounter inaccessible activities, insufficient instructional adaptation, and limited social inclusion, which can constrain their learning experiences and satisfaction (Ruin et al., 2023).

These disability-specific barriers suggest that learning satisfaction cannot be understood solely in terms of general course quality but must also be considered in relation to how instructional and environmental conditions respond to different sensory needs. Understanding these differences is therefore important for developing physical education environments that support autonomy, competence, and relatedness (Ball et al., 2024), while facilitating sustained physical activity engagement, healthy behaviors, and a sense of belonging among students with sensory impairments.

Previous research on physical activity and sport participation among university students with hearing and visual impairments has primarily examined participation patterns, associated health and psychosocial outcomes (Alcaraz-Rodríguez et al., 2022; DeLuca and Rupp, 2022), and participation barriers and facilitators (Barker et al., 2025; Giese and Grenier, 2025; Kurková and Scheetz, 2016; Rich et al., 2024; Ruin et al., 2023). Comparatively little attention has been devoted to learning satisfaction within university physical education. More importantly, it remains unclear which course attributes contribute most strongly to learning satisfaction, which attributes should be prioritized for improvement, and whether these priorities differ between students with hearing and visual impairments. Conventional satisfaction assessments based primarily on attribute ratings can identify favorable or unfavorable evaluations but provide limited guidance regarding the relative contribution of each attribute to overall satisfaction and its priority for improvement (Azzopardi and Nash, 2013; Deng, 2007). RIPA addresses this limitation by deriving attribute importance from its relationship with overall satisfaction and accounting for asymmetric relationships between attribute performance and satisfaction, thereby providing a more informative basis for identifying improvement priorities (Deng, 2007; Deng et al., 2008). Accordingly, the present study compares RIPA priority patterns between university students with hearing and visual impairments to identify both shared and disability-specific priorities for improving physical education learning satisfaction.

Traditional Importance–Performance Analysis (IPA) was developed as a managerial tool for identifying priority areas for improvement (Martilla and James, 1977). However, previous research has indicated that reliance on self-reported importance ratings and mean-based quadrant classifications may lead to unstable attribute categorization, limited explanatory power, and potentially misleading managerial interpretations (Azzopardi and Nash, 2013; Bacon, 2003). To address these limitations, Deng (2007) proposed Revised Importance–Performance Analysis (RIPA), which replaces stated importance with derived importance. Specifically, derived importance is estimated through the partial correlations between attribute performance and overall satisfaction, thereby reflecting the relative contribution of each attribute to overall evaluations. Furthermore, RIPA incorporates three-factor theory to capture the non-linear and asymmetric relationships between attribute performance and satisfaction.

Conceptually, this modification changes the interpretation of “importance” from what respondents explicitly report as important to the extent to which an attribute actually contributes to their overall satisfaction (Bacon, 2003; Deng, 2007). This distinction is particularly relevant for curriculum evaluation because students may report many course attributes as important, whereas these attributes may differ substantially in their actual contribution to overall learning satisfaction (Deng, 2007; Deng et al., 2008). Consequently, RIPA provides a more differentiated basis for distinguishing attributes that require immediate improvement from those that should be maintained or assigned a lower priority (Deng, 2007). From a decision-making perspective, this reduces the risk of allocating limited instructional resources to attributes that students explicitly regard as important but that contribute relatively little to their overall learning satisfaction (Azzopardi and Nash, 2013; Bacon, 2003). Conversely, attributes with high derived importance but relatively low learning satisfaction can be identified as priority areas for intervention (Deng, 2007; Deng et al., 2008). Therefore, RIPA is not merely a technical refinement of traditional IPA; rather, it provides a conceptually different framework for translating students' evaluations into actionable curriculum priorities and resource-allocation decisions (Azzopardi and Nash, 2013; Deng, 2007). This distinction is particularly important in inclusive physical education, where students with different sensory impairments may exhibit different priority patterns across accessibility, instructional support, course content, and learning outcomes. Accordingly, the present study employed RIPA to identify and compare disability-specific priorities in physical education learning satisfaction among university students with visual and hearing impairments. Given the diagnostic and priority-setting nature of RIPA, the study was guided by the following research questions (RQ):

RQ1. Which physical education learning satisfaction attributes are prioritized among university students with visual impairments?

RQ2. Which physical education learning satisfaction attributes are prioritized among university students with hearing impairments?

RQ3. What similarities and differences emerge in the RIPA-derived priority patterns of physical education learning satisfaction attributes between university students with visual and hearing impairments?

## 2 Literature review

### 2.1 Physical education for university students with hearing and visual impairments

Physical education for university students with hearing and visual impairments extends beyond a general physical activity course and serves as an educational context for health promotion, psychological need fulfillment, and equitable participation. According to Self-Determination Theory, learning environments that support autonomy, competence, and relatedness facilitate motivation and adaptive engagement (Ryan and Deci, 2000), a relationship that has also been demonstrated in physical education settings (Standage et al., 2005). Empirical evidence further highlights the value of physical education for students with sensory impairments. Rich et al. (2024) reported that accessible and inclusive sport participation provides physical, psychological, and social benefits for students with hearing impairments. Likewise, Sweeting et al. (2020) found that physical activity interventions for individuals with visual impairments contribute to improvements in mobility, balance, and functional performance. Accordingly, the value of physical education for university students with hearing and visual impairments lies not only in educational outcomes but also in promoting health and supporting sustained engagement in physical activity.

In recent years, research on physical education among university students with hearing impairments has focused primarily on communication accessibility, classroom interaction, and social inclusion (Kurková and Scheetz, 2016; Hardebeck et al., 2026; Rich et al., 2024; Tanure Alves et al., 2021). Rich et al. (2024) found that the experiences of deaf and hard-of-hearing collegiate athletes were strongly influenced by the accessibility and inclusiveness of sport participation environments. Key themes included self-advocacy, meaningful communication, and social connectedness, highlighting the importance of interaction and inclusion in shaping educational experiences. Similarly, Tanure Alves et al. (2021) reported that students with hearing impairments frequently experienced difficulties understanding instructional content and feelings of marginalization within physical education classes. These findings suggest that instructional approaches relying predominantly on verbal communication, without adequate visual support or sign language interpretation, may limit meaningful participation despite students' physical presence in class. Collectively, the evidence indicates that the educational experiences of students with hearing impairments are shaped not only by participation opportunities but also by communication accessibility, social inclusion, and meaningful engagement within the learning environment.

In contrast, research on physical education among students with visual impairments has primarily examined accessibility, safety, social inclusion, and belonging (Haegele et al., 2021; Meier et al., 2023; Ruin et al., 2023). Haegele et al. (2021) found that many students with visual impairments reported limited belonging and inclusion within physical education and were frequently excluded from meaningful participation. Similarly, Ruin et al. (2023) reported that students with visual impairments often experienced social differentiation and hierarchical pressures associated with prevailing notions of normality. These findings suggest that barriers to participation extend beyond instructional adaptation and reflect broader social norms embedded within physical education culture. Furthermore, Meier et al. (2023) noted that previous research has largely examined physical education through the perspectives of teachers, parents, and peers, while the experiences of students with visual impairments have received comparatively limited attention. Consequently, physical education may be perceived as a missed opportunity for developing lifelong physical activity engagement. Accordingly, understanding the needs and experiences of students with visual impairments is essential for designing supportive and inclusive physical education environments that promote sustained participation in physical activity.

Existing research on physical education among students with hearing and visual impairments has focused primarily on educational experiences, inclusion, accessibility, and participation-related outcomes. Although this literature has identified important barriers and support needs, it has largely treated these factors as indicators of participation or educational experience rather than examining how individual course attributes contribute differently to students' learning satisfaction. Learning satisfaction has therefore rarely been conceptualized and examined as a multidimensional construct within inclusive physical education. More importantly, existing approaches provide limited insight into whether an attribute represents a genuine priority for improvement: a relatively unfavorable evaluation may indicate inadequate instructional provision, but it may also concern an attribute that contributes little to students' overall learning satisfaction. Without distinguishing between these possibilities, identifying which aspects of physical education should receive priority becomes difficult, limiting the practical value of existing evidence for curriculum development, instructional adaptation, and resource allocation (Rich et al., 2024). This limitation is particularly relevant when comparing students with hearing and visual impairments, whose barriers and support needs may differ substantially across instructional and environmental contexts.

### 2.2 Learning satisfaction in physical education

Learning satisfaction in university physical education is widely regarded as a multidimensional indicator of educational quality. It reflects students' overall evaluation of the extent to which course content, instructional practices, learning environments, facilities, and assessment approaches meet their needs and expectations. Beyond evaluating instructional effectiveness, learning satisfaction is associated with students' educational experiences, health outcomes, and sustained health behaviors (Jin and Xi, 2026). Jin and Xi (2026) further noted that the development of physical education satisfaction measures aims to enhance learning experiences and promote long-term health behavior adoption. In addition, Chen et al. (2023) found that satisfaction in university physical education was influenced by personality traits, flow experience, and perceived course difficulty, suggesting a close relationship between satisfaction and students' psychological engagement during learning. Accordingly, learning satisfaction should be regarded not merely as an expression of course preference but as an important indicator of the extent to which physical education supports engagement, participation, and sustained health behaviors (Jin and Xi, 2026).

Learning satisfaction is shaped by both instructional interactions and students' psychological engagement. Rather than representing a static evaluation of course arrangements, it reflects students' overall appraisal of instructional quality, course difficulty, personal engagement, and learning experiences. Accordingly, learning satisfaction serves as an important indicator of educational quality in physical education (Jin and Xi, 2026). Supporting this perspective, Cruz and Kim (2023) found that students who perceived higher levels of transformational leadership among physical education instructors reported greater satisfaction with online physical education, increased enjoyment, and stronger perceptions of health and fitness improvement. These findings suggest that learning satisfaction in university physical education is influenced by both instructional practices and psychological engagement. This perspective provides a useful framework for understanding the physical education experiences of university students with disabilities.

Research on learning satisfaction in physical education among university students with disabilities remains limited. Existing studies have primarily examined satisfaction with campus sport facilities, participation in adapted physical activity programs, and institutional support for physical activity engagement rather than learning satisfaction within formal credit-bearing physical education courses. For example, Yoh et al. (2008) reported low levels of facility use and satisfaction among university students with physical disabilities, highlighting persistent accessibility barriers in higher education physical activity environments. Similarly, Dysterheft et al. (2018) and Liska et al. (2024) investigated inclusive aquatic programs and adapted physical activity interventions, respectively, with a focus on participation experiences, motivation, physical activity engagement, and basic psychological needs rather than learning satisfaction. Consequently, current research has emphasized participation-related outcomes and support mechanisms while providing limited insight into students' evaluations of physical education courses. Furthermore, previous studies have not identified which instructional attributes are perceived as most important by university students with disabilities or which require priority improvement. This limitation restricts the identification of key determinants of learning satisfaction and the development of evidence-based recommendations for inclusive physical education.

Based on the preceding literature review, the present study employed RIPA to examine learning satisfaction in physical education among university students with hearing and visual impairments. Rather than treating learning satisfaction as a single overall evaluation, RIPA distinguishes between the relative importance of individual course attributes and students' satisfaction with those attributes, thereby identifying which aspects of physical education warrant priority improvement. Importantly, examining students with hearing and visual impairments separately enables the study to determine whether different sensory-related barriers and learning contexts are associated with distinct priority patterns. Accordingly, this study addresses a gap in the existing literature by moving beyond the identification of general participation barriers and satisfaction levels to establish disability-specific priorities for curriculum improvement. The findings are expected to provide a more differentiated understanding of learning satisfaction and inform the allocation of instructional and support resources in inclusive physical education.

## 3 Method

### 3.1 Research design

This study employed a cross-sectional questionnaire design to examine learning satisfaction in physical education among university students with hearing and visual impairments in mainland China. Universities in Guangdong Province were selected through random sampling, followed by purposive sampling to recruit eligible participants. Data were analyzed using RIPA and three-factor theory to evaluate the relative importance and performance of instructional attributes related to learning satisfaction. The study further compared satisfaction patterns and improvement priorities between students with hearing and visual impairments to inform the development of inclusive physical education practices in higher education.

### 3.2 Research instruments

The Physical Education Learning Satisfaction Scale used in this study was developed with reference to the conceptual definitions and dimensional structures proposed by Tao (2025) and Chen and Xiang (2025), rather than through direct translation or item-by-item adaptation of the original instruments. Accordingly, translation and back-translation procedures were not applicable. Based on these conceptual frameworks, items were developed to reflect the physical education experiences of university students with hearing and visual impairments. The resulting instrument comprised four dimensions: instructional content (4 items), pedagogical methods (4 items), learning outcomes (4 items), and overall experience (4 items). An additional item assessing overall satisfaction with physical education courses was included to derive attribute importance in the RIPA. All items were rated on a seven-point Likert scale ranging from 1 (very dissatisfied) to 7 (very satisfied) (Table 1).

**Table 1:** Physical education learning satisfaction scale.

Variables	Dimensions	Items
Physical education learning satisfaction	Instructional content	1. CT1. The activities selected for this course are appropriate and accessible for students with hearing or visual impairments.
		2. CT2. The course content adequately covers sport skills, physical fitness, and health-related knowledge.
		3. CT3. The difficulty and pace of the course are appropriate for my abilities.
		4. CT4. The instructor clearly explains the objectives and key points of each activity.
	Pedagogical methods	5. MT5. The instructor's explanations, demonstrations, and instructional cues help me understand the required movements.
		6. MT6. When additional explanation or assistance is needed, the instructor provides support patiently.
		7. MT7. The instructor's communication and instructional delivery are clear and easy to follow.
		8. MT8. The instructor adapts teaching methods to meet the needs of students with hearing or visual impairments.
	Learning outcomes	9. OT9. This course has improved my skills and physical fitness.
		10. OT10. This course has increased my understanding of safe exercise and body care.
		11. OT11. This course has increased my confidence in my sports abilities.
		12. OT12. This course has increased my willingness to participate in physical activity outside class.
	Overall experience	13. OE13. I feel treated as an equal member of the class by both the instructor and my classmates.
		14. OE14. The arrangements of the class environment, including facilities, equipment, and group organization, make me feel safe and comfortable.
		15. OE15. Accessibility support provided during physical education classes facilitates my participation.
		16. OE16. My overall experience in this physical education course has been positive and enjoyable.
		17. Overall, I am satisfied with this physical education course.

### 3.3 Data collection

Participant recruitment was conducted using a two-stage sampling procedure. In the first stage, 78 universities in Guangdong Province offering educational programs for students with disabilities constituted the institutional sampling frame. Ten universities were selected from this sampling frame using simple random sampling, with each eligible university having an equal probability of selection. In the second stage, purposive sampling was used to recruit students who met the study eligibility criteria. Participants were eligible if they (1) were currently enrolled university students, (2) had a hearing or visual impairment meeting the specified criteria, (3) had experience participating in university physical education courses, and (4) were able and willing to provide informed consent and complete the questionnaire. Students who did not meet the impairment criteria, had no experience with university physical education courses, or submitted incomplete questionnaires were excluded. Hearing impairment was defined according to World Health Organization (2007) criteria as bilateral hearing loss of at least moderate severity (41–60 dBHL or greater). Visual impairment was defined according to the International Classification of Diseases, 11th Revision (ICD-11; World Health Organization, 2026), as corrected or uncorrected visual acuity below 6/12.

Following university selection, invitation emails describing the study purpose, eligibility criteria, and recruitment procedures were sent to faculty members responsible for disability-related programs at each selected university. After institutional approval, eligible students were identified and invited to participate with the assistance of the responsible faculty members. Participation was voluntary, and no student was required to participate through faculty referral. Paper-based questionnaires were administered on site, and faculty members provided communication or translation assistance when necessary to ensure accessibility without influencing participants' responses. The survey was anonymous, and participants were informed of the study purpose, their rights, the voluntary nature of participation, and the intended use of the data before providing informed consent. Data were collected between August 2025 and January 2026. Although random selection at the university level reduced potential institutional selection bias, purposive recruitment at the participant level may have introduced selection bias because students who were accessible through participating universities and willing to participate may not fully represent all university students with hearing or visual impairments in Guangdong Province. This limitation was considered when interpreting the generalizability of the findings.

A total of 480 questionnaires were collected. After excluding 29 incomplete responses, 449 valid questionnaires were retained for analysis, including 198 students with visual impairments and 251 students with hearing impairments (Table 2). Among students with visual impairments, 102 were male (51.5%) and 96 were female (48.5%). The sample included 53 first-year students (26.8%), 48 second-year students (24.2%), 51 third-year students (25.8%), and 46 fourth-year students (23.2%). Moderate visual impairment was the most prevalent classification (*n* = 103, 52.0%), followed by severe visual impairment (*n* = 57, 28.8%) and total blindness (*n* = 38, 19.2%). Regarding etiology, genetic factors accounted for 25.3% of cases (*n* = 50), followed by retinal diseases (*n* = 44, 22.2%), congenital eye disorders (*n* = 38, 19.2%), optic nerve and other acquired conditions (*n* = 37, 18.7%), and trauma-related causes (*n* = 29, 14.6%).

**Table 2:** Demographic characteristics of respondents.

Disability group	Variables	Category	Frequency	Percentage(%)
Students with visual impairments	Gender	Male	102	51.5
		Female	96	48.5
	Grade	Freshman	53	26.8
		Sophomore	48	24.2
		Junior	51	25.8
		Senior	46	23.2
	Grade of visual impairment	Moderate visual impairment	103	52.0
		Severe visual impairment	57	28.8
		Blindness	38	19.2
	Causes	Genetics	50	25.3
		Congenital eye disease	38	19.2
		Retinal disease	44	22.2
		Trauma	29	14.6
		Optic nerve/other acquired disease	37	18.7
Students with hearing impairments	Gender	Male	135	53.8
		Female	116	46.2
	Grade	Freshman	67	26.7
		Sophomore	61	24.3
		Junior	64	25.5
		Senior	59	23.5
	Grade of hearing impairment	Moderate impairment	145	57.8
		Severe impairment	63	25.1
		Profound impairment	43	17.1
	Causes	Genetics	71	28.3
		Ear infection	31	12.4
		Chemical exposure	24	9.6
		Long-term noise exposure	54	21.5
		Trauma	71	28.3

Among students with hearing impairments, 135 were male (53.8%) and 116 were female (46.2%). The sample included 67 first-year students (26.7%), 61 second-year students (24.3%), 64 third-year students (25.5%), and 59 fourth-year students (23.5%). Moderate hearing impairment was the most prevalent classification (*n* = 145, 57.8%), followed by severe hearing impairment (*n* = 63, 25.1%) and profound hearing impairment (*n* = 43, 17.1%). Regarding etiology, genetic factors and traumatic injury each accounted for 28.3% of cases (*n* = 71), followed by long-term noise exposure (*n* = 54, 21.5%), ear infections (*n* = 31, 12.4%), and chemical exposure (*n* = 24, 9.6%).

Sample size requirements for CFA depend on model characteristics, including the number of factors and indicators, factor loadings, and model complexity, rather than on a single universal threshold (Wolf et al., 2013). The present study included 449 participants, comprising 251 students with hearing impairments and 198 students with visual impairments. Given the 16-item, four-factor measurement model and the satisfactory confirmatory factor analysis (CFA) results obtained separately for both groups, the sample sizes were considered adequate for the measurement analyses and subsequent RIPA.

### 3.4 Data analysis

The collected data were analyzed using IBM SPSS Statistics and SmartPLS 3.0. CFA (CB-SEM) was conducted to evaluate the reliability and validity of the learning satisfaction scale. RIPA was subsequently employed to assess the relative importance and performance of learning satisfaction attributes among students with visual and hearing impairments.

### 3.5 Ethical considerations

This study was reviewed and approved by the Academic Committee of the School of Physical Education and Sports Science at Lingnan Normal University (Protocol No. LNU20260509; Project No. ZL2021008) prior to data collection. All participants were informed of the study purpose, research procedures, their rights as participants, and data confidentiality. Written informed consent was obtained before questionnaire administration, and all responses were collected anonymously.

## 4 Results

The reliability and validity of the Physical Education Learning Satisfaction Scale were evaluated among university students with visual impairments. The four-factor model demonstrated a good overall fit to the data (χ^2^/df = 1.335, CFI = 0.989, TLI = 0.986, RMSEA = 0.041, 90% CI [0.019, 0.059], and SRMR = 0.035), providing empirical support for the proposed dimensional structure beyond the evaluation of individual factor loadings. The measurement model demonstrated acceptable psychometric properties (Tables 3, 4). Factor loadings ranged from 0.786 to 0.926, indicating strong relationships between the observed indicators and their respective constructs. Cronbach's α and composite reliability (CR) values ranged from 0.897 to 0.955, exceeding recommended thresholds for internal consistency. McDonald's ω values ranged from 0.897 to 0.955, providing additional evidence of internal consistency without relying on the assumption of tau-equivalence. The average variance extracted (AVE) ranged from 0.685 to 0.843, supporting convergent validity. Furthermore, the square root of AVE for each construct (0.827–0.918) exceeded the corresponding inter-construct correlations (0.406–0.524), indicating adequate discriminant validity. Taken together, the model fit, substantial factor loadings, internal consistency, convergent validity, and discriminant validity indicate that the items collectively form a coherent four-dimensional representation of physical education learning satisfaction among university students with visual impairments.

**Table 3:** Reliability and validity of the physical education learning satisfaction scale for university students with visual impairments.

Variable	Dimensions	Items	Factor loading	Cronbach's α	C.R.	AVE	McDonald's ω
Physical education learning satisfaction	Instructional content	CT1	0.926	0.955	0.955	0.843	0.955
		CT2	0.912				
		CT3	0.911				
		CT4	0.923				
	Pedagogical methods	MT5	0.906	0.947	0.947	0.817	0.947
		MT6	0.918				
		MT7	0.890				
		MT8	0.901				
	Learning outcomes	OT9	0.882	0.929	0.929	0.766	0.929
		OT10	0.880				
		OT11	0.882				
		OT12	0.856				
	Overall experience	OE13	0.849	0.897	0.897	0.685	0.897
		OE14	0.825				
		OE15	0.848				
		OE16	0.786				
χ^2^/df	1.335						
CFI	0.989						
TLI	0.986						
RMSEA	0.041						
CI	0.019, 0.059						
SRMR	0.035						

**Table 4:** Discriminant validity of the physical education learning satisfaction scale among university students with visual impairments.

Variable	CT	MT	OE	OT
CT	0.918			
MT	0.444	0.904		
OE	0.406	0.481	0.827	
OT	0.406	0.440	0.524	0.875

CT, Instructional content; MT, Pedagogical methods; OT, Learning outcomes; OE, Overall experience.

The reliability and validity of the Physical Education Learning Satisfaction Scale were evaluated among university students with hearing impairments. The four-factor model demonstrated a good overall fit to the data (χ^2^/df = 1.039, CFI = 0.999, TLI = 0.999, RMSEA = 0.013, 90% CI [0.000, 0.036], and SRMR = 0.023), providing empirical support for the proposed dimensional structure beyond the evaluation of individual factor loadings. The measurement model exhibited satisfactory psychometric properties (Tables 5, 6). Factor loadings ranged from 0.814 to 0.928, while Cronbach's α and CR values ranged from 0.896 to 0.954, indicating strong indicator reliability and internal consistency. McDonald's ω values ranged from 0.896 to 0.953, providing additional evidence of internal consistency without relying on the assumption of tau-equivalence. AVE values ranged from 0.684 to 0.837, supporting convergent validity. In addition, the square root of AVE for each construct (0.827–0.915) exceeded the corresponding inter-construct correlations (0.455–0.555), satisfying the criterion for discriminant validity. Taken together, the model fit, substantial factor loadings, internal consistency, convergent validity, and discriminant validity indicate that the items collectively form a coherent four-dimensional representation of physical education learning satisfaction among university students with hearing impairments.

**Table 5:** Reliability and validity of the physical education learning satisfaction scale among university students with hearing impairments.

Variable	Dimensions	Items	Factor loading	Cronbach's α	C.R.	AVE	McDonald's ω
Physical education learning satisfaction	Instructional content	CT1	0.	0.953	0.954	0.837	0.953
		CT2	0.928				
		CT3	0.910				
		CT4	0.903				
	Pedagogical methods	MT5	0.	0.945	0.945	0.812	0.945
		MT6	0.917				
		MT7	0.894				
		MT8	0.885				
	Learning outcomes	OT9	0.	0.930	0.930	0.769	0.930
		OT10	0.905				
		OT11	0.851				
		OT12	0.860				
	Overall experience	OE13	0.	0.896	0.896	0.684	0.896
		OE14	0.844				
		OE15	0.814				
		OE16	0.822				
χ^2^/df	1.039						
CFI	0.999						
TLI	0.999						
RMSEA	0.013						
CI	0.000, 0.036						
SRMR	0.023						

**Table 6:** Discriminant validity of the physical education learning satisfaction scale among university students with hearing impairments.

Variable	CT	MT	OE	OT
CT	0.915			
MT	0.455	0.901		
OE	0.481	0.519	0.827	
OT	0.501	0.516	0.555	0.877

CT: Instructional content; MT: Pedagogical methods; OT: Learning outcomes; OE: Overall experience.

Potential common method bias was assessed separately for students with visual and hearing impairments using Harman's single-factor test. All 16 measurement items were simultaneously entered into an unrotated factor analysis. As shown in Table 7, the first factor accounted for 46.85% of the total variance among students with visual impairments, indicating that no single factor explained the majority of the variance. Among students with hearing impairments, the first factor accounted for 50.12% of the total variance, which was marginally above the commonly applied 50% reference value. Thus, common method variance was not evident as a dominant concern in the visual-impairment sample, whereas its potential influence in the hearing-impairment sample cannot be entirely excluded. Given the marginal nature of this result, the findings for students with hearing impairments should be interpreted with appropriate caution.

**Table 7:** Results of Harman's single-factor test.

Groups	Sample size (*n*)	Number of Items	Variance explained by the first factor (%)	Assessment
Students with visual impairments	198	16	46.85	Below 50%
Students with hearing impairments	251	16	50.12	Marginally above 50%

**Table 8:** Cross-group comparison of RIPA results.

RIPA quadrant	Visual impairments	Hearing impairments	Key comparison
Keep up the good work	CT2, OT9	CT2, CT4, MT6	CT2 is the only shared strength.
Concentrate here	OT12	OT9, OT12, MT8, OE14, OE15	OT12 is the only shared priority; hearing-impaired students show broader improvement needs.
Possible overkill	CT1, CT3, CT4, MT5, MT6, MT7	CT1, CT3, MT5	CT1, CT3, and MT5 are shared; CT4 and MT6 shift to strengths in the hearing group.
Low priority	MT8, OT10, OT11, OE13, OE14, OE15, OE16	MT7, OT10, OT11, OE13, OE16	MT8, OE14, and OE15 shift from Low Priority to Concentrate Here in the hearing group.

### 4.1 RIPA analysis for university students with visual impairments

RIPA was employed to examine the distribution of learning satisfaction attributes in physical education among university students with visual impairments (Figure 1). The “Keep Up the Good Work” quadrant included CT2 (course content integration) and OT9 (perceived improvement in sport ability and physical or psychological relaxation), indicating that these attributes were characterized by high importance and high performance. These findings suggest that course content and perceived learning outcomes represent key strengths of the current curriculum. In contrast, the “Concentrate Here” quadrant contained only OT12 (awareness of sustained exercise participation), indicating that although students regarded continued engagement in physical activity as highly important, the curriculum was less effective in fostering this outcome. Accordingly, OT12 represents a priority area for curriculum improvement.

**Figure 1:**
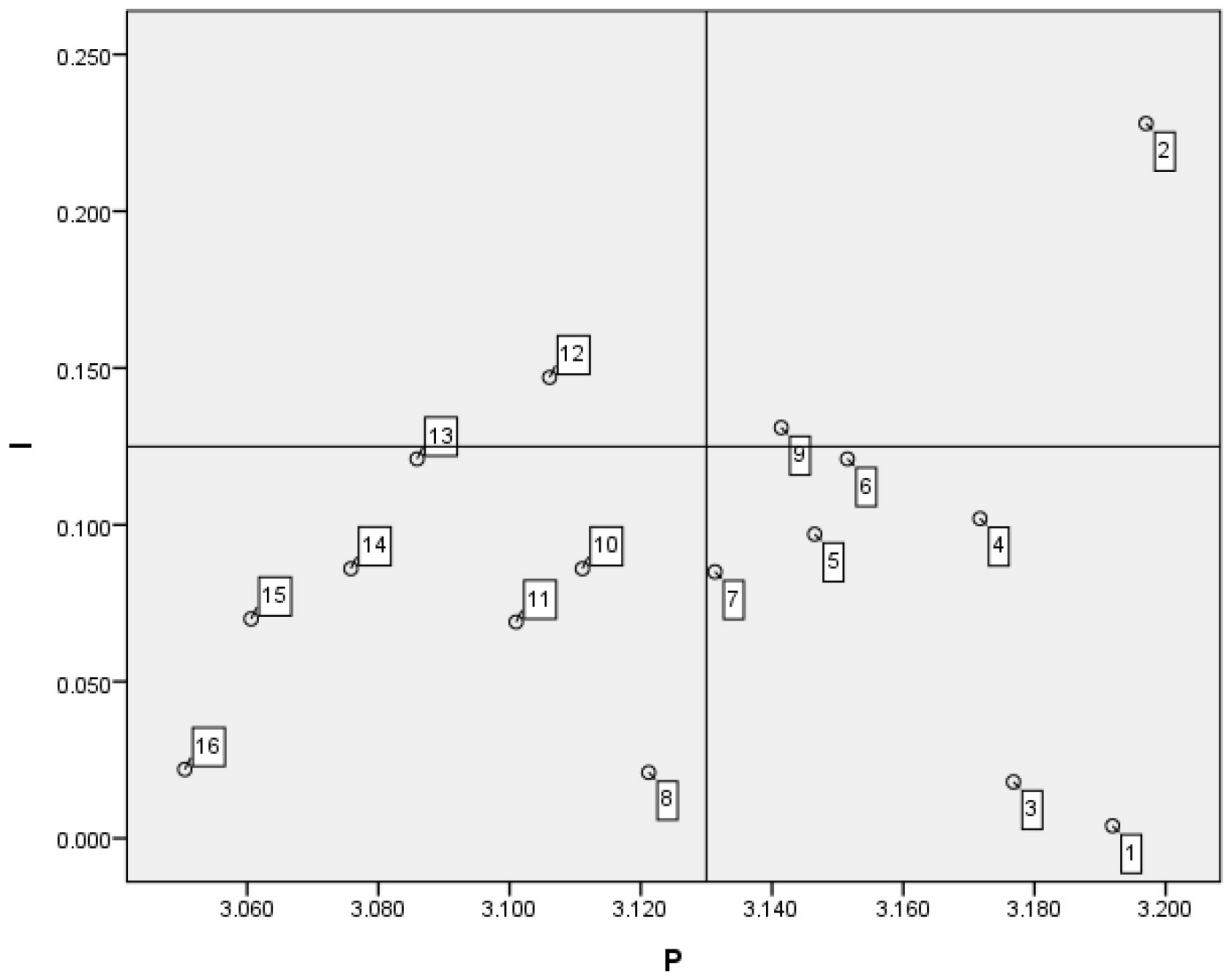
The perceptual map of students with visual impairments.

The “Possible Overkill” quadrant included CT1 (appropriateness of sport activities), CT3 (appropriateness of course difficulty), CT4 (clarity of activity explanations), MT5 (support for movement comprehension), MT6 (individualized instructional support), and MT7 (clarity of instructional delivery). These attributes were characterized by relatively high performance but comparatively lower importance, suggesting that they contributed less to overall learning satisfaction despite favorable evaluations. By contrast, the “Low Priority” quadrant included MT8 (flexibility in teaching adjustment), OT10 (understanding of safe exercise knowledge), OT11 (improvement in exercise confidence), OE13 (perceived equal treatment), OE14 (sense of activity safety), OE15 (accessibility support), and OE16 (overall emotional experience). These attributes exhibited relatively low levels of both importance and performance, indicating a limited contribution to overall learning satisfaction. Nevertheless, their placement may inform future curriculum refinement and the continued development of accessible physical education practices.

Overall, the RIPA results indicated that learning satisfaction among university students with visual impairments was primarily associated with integrated course content and positive post-class experiences. In contrast, awareness of sustained exercise participation emerged as the principal area requiring improvement, reflecting high importance but comparatively low performance. These findings suggest the need for greater emphasis on instructional strategies that encourage continued engagement in physical activity beyond the classroom. Such efforts may strengthen the capacity of physical education courses to support sustained participation and long-term health promotion among university students with visual impairments.

### 4.2 RIPA analysis for university students with hearing impairments

The RIPA results for learning satisfaction in physical education among university students with hearing impairments are presented in Figure 2. The “Keep Up the Good Work” quadrant included CT2 (course content integration), CT4 (clarity of activity explanations), and MT6 (individualized instructional support), indicating high levels of both importance and performance. These attributes represent key strengths of the current physical education curriculum. In contrast, the “Concentrate Here” quadrant comprised OT9 (post-class relaxation), OT12 (awareness of sustained exercise participation), MT8 (flexibility in teaching adjustment), OE14 (sense of activity safety), and OE15 (accessibility support). The placement of these attributes indicates high importance but comparatively lower performance, suggesting that post-class experiences, sustained physical activity participation, instructional adaptability, and accessibility support constitute priority areas for improvement.

**Figure 2:**
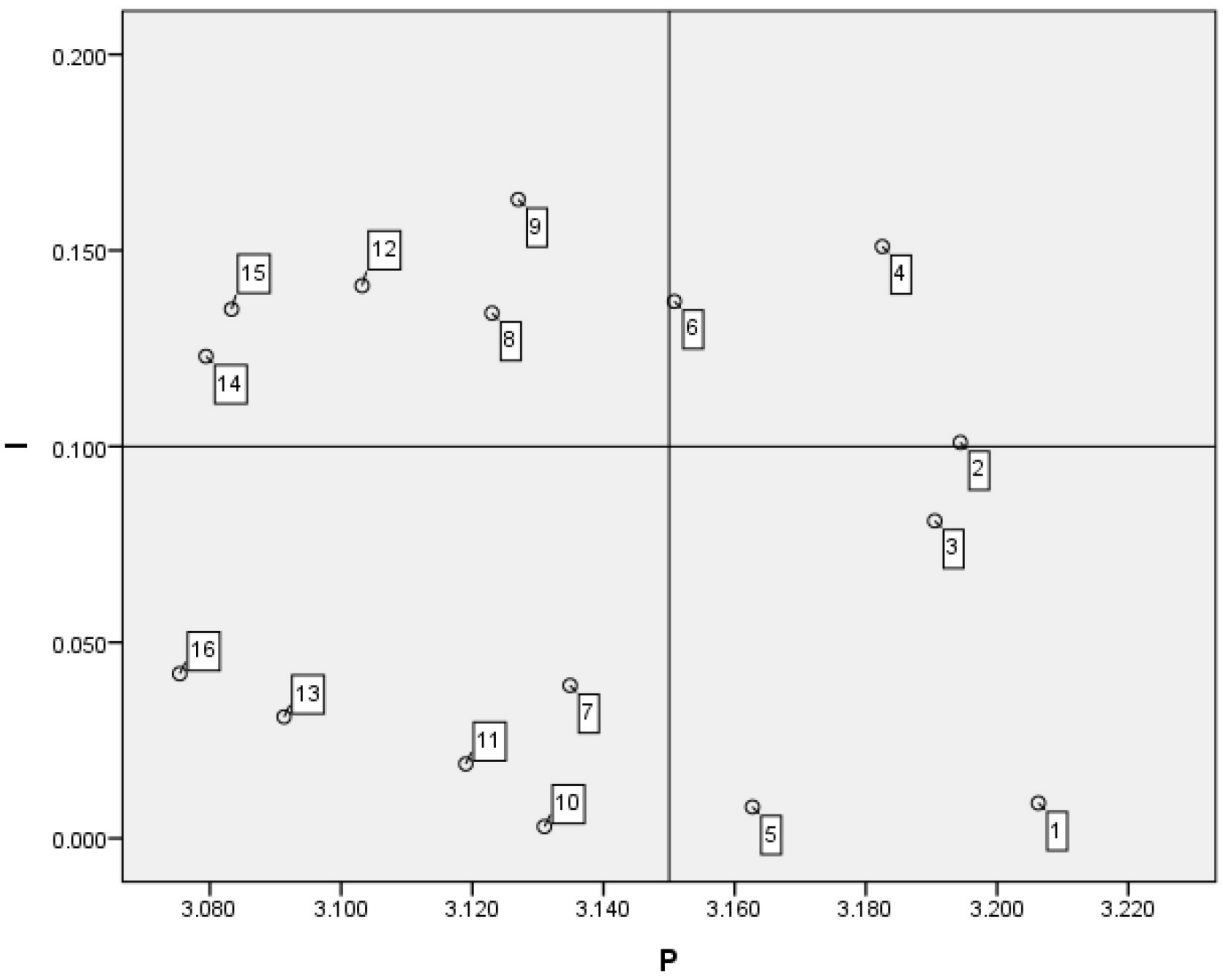
The perceptual map of students with hearing impairments.

The “Possible Overkill” quadrant included CT1 (appropriateness of sport activities), CT3 (appropriateness of course difficulty), and MT5 (support for movement comprehension). These attributes exhibited relatively high performance but comparatively lower importance, suggesting a limited contribution to overall learning satisfaction despite favorable evaluations. By contrast, the “Low Priority” quadrant comprised MT7 (clarity of instructional delivery), OT10 (understanding of safe exercise knowledge), OT11 (improvement in exercise confidence), OE13 (perceived equal treatment), and OE16 (overall emotional experience). These attributes were characterized by relatively low levels of both importance and performance, indicating a comparatively minor influence on overall learning satisfaction. Nevertheless, their placement may inform future instructional refinement and curriculum development.

Overall, the RIPA findings indicated that learning satisfaction among university students with hearing impairments was primarily associated with integrated course content and instructional support. By contrast, awareness of sustained exercise participation, instructional adaptability, accessibility support, and post-class experiences emerged as key areas for improvement, reflecting high importance but comparatively low performance. These findings suggest the need for greater emphasis on instructional and support strategies that facilitate continued engagement in physical activity and accessible participation. Such efforts may strengthen the health-promoting function of physical education and support sustained physical activity participation among university students with hearing impairments.

### 4.3 Cross-group comparison of RIPA-derived priority patterns

A cross-group comparison of the RIPA results revealed both shared and distinct patterns of learning satisfaction between students with visual and hearing impairments (Table 7). CT2 (course content integration) was the only attribute classified as “Keep Up the Good Work” for both groups, indicating a common strength of the current physical education curriculum. In contrast, OT12 (awareness of sustained exercise participation) was the only shared attribute in the “Concentrate Here” quadrant, suggesting that promoting continued engagement in physical activity represents a common challenge for both groups.

Several notable differences were also observed. Among students with visual impairments, OT9 (post-class relaxation) was identified as a strength, whereas students with hearing impairments showed additional strengths in CT4 (clarity of activity explanations) and MT6 (individualized instructional support). More importantly, MT8 (flexibility in teaching adjustment), OE14 (sense of activity safety), and OE15 (accessibility support) were classified as “Low Priority” for students with visual impairments but as “Concentrate Here” for students with hearing impairments. Similarly, CT4 and MT6 shifted from “Possible Overkill” among students with visual impairments to “Keep Up the Good Work” among students with hearing impairments. These differences indicate that instructional adaptability, accessibility, and instructional support contribute differently to learning satisfaction across the two groups.

Overall, the comparison indicates that students with sensory impairments should not be treated as a homogeneous group in physical education. Although integrated course content and sustained exercise participation represent shared concerns, the two groups differ in the relative importance and learning satisfaction associated with instructional and accessibility-related attributes. These findings demonstrate the value of RIPA in identifying disability-specific priorities and suggest that differentiated rather than uniform strategies may be more appropriate for improving inclusive physical education.

## 5 Discussion

The RIPA findings revealed that several attributes emphasized in previous research, including accessibility support, equal treatment, and perceived safety, did not emerge as high-priority determinants of learning satisfaction. This finding appears to differ from previous studies that identified accessibility, safety, and inclusion as central concerns for students with visual impairments (Haegele et al., 2021; Ruin et al., 2023). In particular, MT8 (flexibility in teaching adjustment), OE14 (sense of activity safety), and OE15 (accessibility support) were classified as “Low Priority” among students with visual impairments. This does not imply that these attributes are unimportant; rather, their derived importance to overall learning satisfaction was comparatively low in the present context. From the perspective of Self-Determination Theory, students' evaluations of learning environments depend on the extent to which their needs for autonomy, competence, and relatedness are satisfied (Ryan and Deci, 2000). When basic conditions such as safety and accessibility are sufficiently provided, they may be perceived as expected features rather than salient determinants of satisfaction. Thus, the present findings extend previous research by suggesting that the contribution of accessibility and safety to learning satisfaction may depend on the extent to which these needs are already addressed within the educational environment.

More importantly, the RIPA results revealed distinct priority patterns between the two groups. MT8, OE14, and OE15 shifted from “Low Priority” among students with visual impairments to “Concentrate Here” among students with hearing impairments. Similarly, CT4 (clarity of activity explanations) and MT6 (individualized instructional support) shifted from “Possible Overkill” to “Keep Up the Good Work.” These differences may reflect the persistent communication and instructional-access barriers experienced by students with hearing impairments (Kurková and Scheetz, 2016; Tanure Alves et al., 2021), making instructional adaptability and accessibility more influential in their learning satisfaction. In contrast, students with visual impairments often encounter barriers related to spatial navigation, activity accessibility, and movement control (Ruin et al., 2021). When these immediate participation needs are adequately addressed, their evaluation may shift toward longer-term outcomes (Haegele et al., 2022). This may explain why OT12 (awareness of sustained exercise participation) emerged as the only priority for students with visual impairments. Notably, OT12 was also a priority among students with hearing impairments, suggesting that translating physical education experiences into sustained physical activity remains a common challenge across both groups.

These findings also illustrate the added value of RIPA. Learning satisfaction is multidimensional and shaped by instructional, psychological, and contextual factors (Chen et al., 2023; Jin and Xi, 2026); therefore, low satisfaction alone does not necessarily indicate a high-priority area for intervention. By jointly considering derived importance and learning satisfaction, RIPA distinguishes attributes that require immediate improvement from those that receive relatively low satisfaction but contribute less to overall learning satisfaction. The contrasting quadrant positions across the two groups further demonstrate that students with sensory impairments should not be treated as a homogeneous population. For students with visual impairments, the primary priority concerns sustained exercise participation, whereas students with hearing impairments require broader attention to sustained engagement, instructional adaptability, safety, and accessibility support. Accordingly, RIPA provides both an analytical and decision-making framework for identifying disability-specific priorities and guiding the allocation of instructional resources.

### 5.1 Theoretical contributions

The primary theoretical contribution of the present study lies in the application of RIPA to the investigation of learning satisfaction in physical education among university students with sensory impairments. This approach extends previous research, which has largely focused on qualitative experiences, participation barriers, or overall satisfaction outcomes as primary analytical frameworks. Prior studies on physical education among students with hearing and visual impairments have primarily emphasized accessibility, inclusivity, communication barriers, and social participation (Haegele et al., 2020; Kurková and Scheetz, 2016; Rich et al., 2024). However, relatively limited attention has been given to distinguishing between the course attributes that students perceive as important and their actual levels of learning satisfaction. Consequently, although previous studies have identified the challenges encountered by students, they have provided limited insight into which issues should be prioritized for improvement.

By incorporating both perceived importance and performance, RIPA enabled learning satisfaction in physical education to be examined as a multidimensional construct rather than a single outcome measure. This approach provides a more systematic basis for identifying priority needs and informing curriculum development within inclusive physical education contexts. The findings revealed distinct patterns of learning priorities between students with visual and hearing impairments, despite their shared classification as students with sensory impairments. These differences suggest that learning satisfaction is shaped by disability-specific challenges and educational contexts, rather than by sensory impairment alone. This interpretation is consistent with Self-Determination Theory, which emphasizes the role of contextual support in satisfying individuals' needs for autonomy, competence, and relatedness (Ryan and Deci, 2000). Accordingly, the present study extends existing research on learning satisfaction in physical education, which has largely focused on general university student populations (Chen et al., 2023; Jin and Xi, 2026), by demonstrating the heterogeneous nature of learning needs among students with sensory impairments.

In addition, the present study contributes to the theoretical development of adapted physical education research. Previous studies have largely emphasized participation as a primary indicator of successful inclusion. The present findings suggest that, for university students with sensory impairments, learning satisfaction is influenced not only by opportunities for participation but also by the extent to which physical education supports sustained exercise awareness, positive psychological experiences, and long-term health-related engagement. From this perspective, the value of physical education extends beyond immediate participation outcomes to its capacity to promote sustained health behaviors and continued involvement in physical activity. Accordingly, the findings broaden existing perspectives in adapted physical education and disability research by highlighting the importance of long-term developmental outcomes alongside participation.

## 6 Implications

The findings of the present study offer important implications for curriculum development and accessible teaching practices in higher education. First, the results indicate that students with visual and hearing impairments exhibit distinct patterns of learning needs, suggesting that a uniform instructional approach may be insufficient within inclusive physical education settings. Instead, instructional support should be tailored to disability-specific characteristics and learning contexts. For students with visual impairments, integrated course content and positive post-class experiences emerged as key strengths, whereas sustained exercise participation was identified as the primary area for improvement. Accordingly, curriculum design should extend beyond the provision of safe and accessible learning environments to include strategies that promote continued engagement in physical activity, such as exercise planning, self-management skills, and autonomous participation beyond formal coursework. Such efforts may strengthen the long-term health-promoting function of physical education.

In contrast, students with hearing impairments assigned greater importance to instructional clarity, individualized support, instructional adaptability, and accessibility-related assistance. These findings highlight the importance of communication-accessible teaching practices in physical education. Accordingly, instructors may enhance learning experiences by incorporating visual instructional materials, non-verbal communication strategies, real-time demonstrations, and individualized guidance to facilitate content comprehension and reduce communication-related barriers. Furthermore, flexible instructional arrangements and appropriate support mechanisms may strengthen students' engagement and learning satisfaction by improving accessibility and participation within the learning environment.

The findings suggest that RIPA provides a useful framework for informing resource allocation and curriculum development in higher education physical education. Traditional approaches to curriculum evaluation have relied largely on overall satisfaction as an indicator of instructional effectiveness. The present findings demonstrate that satisfaction and importance do not necessarily correspond, highlighting the limitations of relying exclusively on satisfaction-based evaluations. Accordingly, curriculum improvement efforts should consider both the relative importance of course attributes and students' evaluations of their performance. Such an approach may facilitate more effective prioritization of instructional resources and support evidence-based decision-making in curriculum design.

Finally, the findings have important implications for accessibility policy in higher education. Physical education provision for students with sensory impairments should extend beyond ensuring access and participation to encompass learning quality, health promotion, and sustained engagement in physical activity. From this perspective, accessibility should be understood not only as a condition for participation but also as a mechanism for supporting meaningful learning experiences and long-term developmental outcomes. Accordingly, universities may strengthen the educational value of physical education through adapted support services, accessibility-oriented teacher preparation, and inclusive curriculum design.

### 6.1 Limitations of the study

The present study is subject to several limitations. First, the cross-sectional design captured learning satisfaction and RIPA perceptual distributions among university students with visual and hearing impairments within a specific study period. Consequently, causal relationships between physical education experiences, physical activity participation, health-related outcomes, and sustained engagement cannot be established. Future research should employ longitudinal designs to examine how physical education experiences and learning satisfaction influence physical activity behaviors and health-related outcomes over time.

Second, the study relied on self-reported data, which may be subject to social desirability and response biases as well as individual differences in subjective evaluation. Students may also evaluate their physical education experiences relative to previous opportunities for participation and support, resulting in different expectations and reference standards for assessing attribute importance and learning satisfaction. Future studies should incorporate multiple data sources, such as qualitative interviews, classroom observations, and instructor perspectives, to provide a more comprehensive assessment of students' physical education experiences.

Third, although the study compared students with visual and hearing impairments, potentially important within-group differences, including impairment severity, assistive technology use, inclusive education experience, and prior sport participation, were not examined. In addition, participants were recruited from 10 universities in Guangdong Province, which may limit the generalizability of the findings to students with sensory impairments in other universities, regions, or educational contexts. The findings should therefore be interpreted with consideration of both within-group heterogeneity and the characteristics of the sampled institutions. Future research should include more geographically and institutionally diverse samples and examine relevant individual characteristics to provide a more nuanced understanding of learning satisfaction and improvement priorities in inclusive physical education.

## 7 Conclusion

The present study employed RIPA to examine learning satisfaction in physical education among university students with visual and hearing impairments and to compare differences in learning priorities across sensory disability groups. The findings indicate that the needs of students with sensory impairments are shaped by both disability-specific characteristics and educational context, resulting in distinct patterns of learning satisfaction and improvement priorities. While students with visual impairments placed greater emphasis on integrated course content and post-class experiences, students with hearing impairments assigned greater importance to instructional support, communication accessibility, and instructional adaptability. The findings further demonstrate that attributes traditionally regarded as important do not necessarily constitute the highest priorities for improvement, highlighting the context-dependent nature of learning satisfaction. By incorporating both perceived importance and performance, the present study extends existing research on adapted physical education and provides a more differentiated understanding of learning satisfaction among students with sensory impairments. The priority attributes identified through RIPA offer practical guidance for curriculum development, accessible instructional design, and resource allocation in higher education, thereby supporting more inclusive and effective physical education environments.

## Data Availability

The datasets presented in this article are not readily available because the dataset is not publicly available because it contains information collected from students with disabilities and is subject to ethical and privacy restrictions. De-identified data may be made available from the corresponding author upon reasonable request and with approval from the relevant ethics committee. Requests to access the datasets should be directed to zhanwp2024@163.com.
